# MRI and Ultrasound Analysis of Corticosteroid Injection Combined with Extracorporeal Shockwave Therapy in Lateral Epicondylitis—A Prospective, Randomized, Double-Blinded, Placebo-Controlled Trial

**DOI:** 10.3390/jpm12111892

**Published:** 2022-11-11

**Authors:** Gopal Nambi, Mshari Alghadier, Elturabi Elsayed Ebrahim, Anju Verma, Osama R. Aldhafian, Shahul Hameed Pakkir Mohamed, Shady Abdullah Alshewaier, Mohamed Sherif Sirajudeen, Hariraja Muthusamy, Radhakrishnan Unnikrishnan

**Affiliations:** 1 Department of Health and Rehabilitation Sciences, College of Applied Medical Sciences, Prince Sattam bin Abdulaziz University, Al-Kharj 11942, Saudi Arabia; 2 Department of Nursing, College of Applied Medical Sciences, Prince Sattam bin Abdulaziz University, Al-Kharj 11942, Saudi Arabia; 3 Department of Exercise and Sports Sciences, School of Health Sciences, Faculty of Medicine and Health, University of Sydney, Camperdown, NSW 2050, Australia; 4 Department of Surgery, College of Medicine, Prince Sattam bin Abdulaziz University, Al-Kharj 11942, Saudi Arabia; 5 Department of Physical Therapy, Faculty of Applied Medical Sciences, University of Tabuk, Tabuk 71491, Saudi Arabia; 6 Department of Physical Therapy and Health Rehabilitation, College of Applied Medical Sciences, Majmaah University, Majmaah 11952, Saudi Arabia

**Keywords:** corticosteroid injection, extracorporeal shockwave therapy, lateral epicondylitis, placebo

## Abstract

Objective: The knowledge about the effective implementation of corticosteroid injection and extracorporeal shockwave therapy on radiological changes in chronic lateral epicondylitis is lacking. Therefore, the objective of this study is to find and compare the effects of corticosteroid injection and extracorporeal shockwave therapy on radiological changes in chronic lateral epicondylitis. Methods: A randomized, double-blinded controlled study was conducted on 60 LE participants at a university hospital. The active extracorporeal shockwave therapy group (*n* = 30) received a corticosteroid injection with active extracorporeal shockwave therapy one session a week for 4 weeks, and the placebo extracorporeal shockwave therapy group received a corticosteroid injection with placebo extracorporeal shockwave therapy. The primary outcome was pain intensity, measured with the visual analog scale. The other outcome measures were the percentage of injury measured by magnetic resonance imaging and ultrasound, functional disability, handgrip strength, patient perception, kinesiophobia, depression status, and quality of life. Results: The between-group difference in pain intensity at 4 weeks was 1.4 (CI 95% 0.77 to 2.02), which shows more improvement in the active group than in the placebo group. Improvements in the effects were noted after 8 weeks and at 6 months (1.8; CI 95% 1.50 to 2.09) follow-up. Similar improvements were also found in the percentage of injury, functional disability, handgrip strength, patient perception, kinesiophobia, depression status, and quality of life. Conclusion: Extracorporeal shockwave therapy has added effects on corticosteroid injection for improving pain, percentage of injury, functional disability, handgrip strength, patient perception, kinesiophobia, depression status and quality of life in people with chronic lateral epicondylitis.

## 1. Introduction

Lateral epicondylitis (LE) is a common musculoskeletal condition with clinical symptoms of pain and inflammation over the proximal attachment of the wrist extensor tendon and lateral epicondyle of the humerus. It is frequently known as “tennis elbow” and maximum incidences occur between 30 and 65 years of age [1]. Other symptoms may include stiffness or weakness in the elbow and difficulties in functional activities of the hand [2]. It is caused by a direct injury or repeated stress or motion of the soft tissues surrounding the elbow joint. Over time, this repeated stress and overloading can cause a degenerative condition known as tendinosis. Together, tendinitis and tendinosis further lead to the tendon tearing. Different conservative, medical, and surgical treatments are available for the treatment of this condition. Amongst these, corticosteroid injection and physical therapy approaches are widely used in the earlier stages of lateral epicondylitis. Studies show the superior effect of corticosteroid injection in LE [3,4], but it is contradicted by some studies [5,6]. It has been observed that steroid injection alone as the first line of treatment for patients presenting with tennis elbow demands a quick return to daily activities by suppressing the granulomatous response in traumatized tissue [7]. The early response of corticosteroids may be due to an analgesic effect on the neuropeptides, calcitonin gene-related peptide, and substance P, which are increased in tendinopathy [6]. Moreover, so far, it has not been clinically proven that these changes are clinically helpful or harmful to LE patients.

In recent times, corticosteroid injections are being discouraged by many physicians because of the high recurrence rate, despite showing a marked short-term effect [5,8]. Recurrence may occur because corticosteroids do not address the key features of tendinopathy, which is traditionally thought to be associated with overuse, cumulative trauma weakening the collagen cross-linking, and the non-collagenous matrix and vascular elements of the tendon. Corticosteroids might be deleterious to the tendon through an effect on fibroblasts’ role in collagen and extracellular matrix protein production [6]. From a clinical perspective, orthopedic surgeons encourage physical therapy in combination with corticosteroid injection for lateral epicondylitis. Physical therapy treatments offered for LE include rest, ice, hydrocolloid packs, infrared radiation, short-wave diathermy, ultrasound, laser, physical exercises, and splint or brace application. A study by Newcomer et al. showed no significant changes in the outcome when an injection is combined with physical therapy for patients with LE on a short-term basis [9]. Extracorporeal shockwave therapy (ESWT) is one of the most successful treatment modalities used in physical therapy; some studies investigated the effect of shockwave therapy in patients with tennis elbow, with a success rate ranging from 68% to 91% [10,11,12].

Along with the regular investigation procedures, there is a need to find the radiological changes after corticosteroid injection with shockwave therapy in lateral epicondylitis. Advanced radio imaging techniques such as magnetic resonance imaging (MRI) and ultrasonography (US) have been used to find the extent of disease and to measure the degree of injury [13,14]. So far, no studies have been conducted to find the radiological changes after corticosteroid injection with ESWT in treating lateral epicondylitis. Therefore, this study aims to investigate the MRI and ultrasound (US) changes after corticosteroid injection with shockwave therapy in lateral epicondylitis. Radiological analysis through MRI and US will provide real-time changes in the soft tissues after different interventions, which will provide sound evidence for the therapists and clinicians to select an optimum intervention for lateral epicondylitis.

## 2. Materials and Methods

### 2.1. Trial Design

This trial was a prospective, randomized, parallel-group, placebo-controlled trial executed at the Department of Physical Therapy, Prince Sattam bin Abdulaziz University, Al-Kharj, Saudi Arabia. The participants were recruited between 1 May 2020 and 1 January 2022. This study was designed and conducted according to the principles of the Declaration of Helsinki and was approved by the Department Ethics Committee with an ethical approval number of RHPT/020/011. The trial was registered prospectively in ClinicalTrial.gov.in with the registration number CTRI/2020/04/024730 on 20 April 2020.

### 2.2. Participants

Male participants aged between 18 and 60 years who were referred to an outpatient physiotherapy clinic with a clinical diagnosis of chronic (pain for more than two months) lateral epicondylitis (M77.1 in ICD-10—International Classification of Diseases) with a pain intensity of 3 to 8 on the visual analog scale (VAS) were invited to participate. It was noted that male participants recorded a higher risk of injury manifestation (68.10%) than female participants, which may alter the study reports. Participants who had received prior corticosteroid injection therapy, had associated neck or arm pain, severe musculoskeletal, neural, somatic and psychiatric conditions, were waiting for surgery, abused alcohol or drugs, or were involved in other weight-training programs were excluded from the study. Participants with other soft tissue injuries, fractures in the limbs, and deformities were also excluded from the study.

All of the participants received a referral letter from the referring hospital to participate in the trial. After signing the written informed consent form and before baseline evaluation, the participants were randomized using a computer-generated randomization method and allocated into two groups: corticosteroid injection with ESWT—active group (*n* = 30) and corticosteroid injection with ESWT—placebo group (*n* = 30). The participants were allocated by a physical therapy assistant through an on-site computer system in which allocation was concealed. Each participant’s group allocation was only revealed to the physical therapist who provided the treatment immediately before the first intervention. The participants were not aware of which treatment they were receiving (blind participants); however, they were informed that they would receive one of the two interventions. Due to the nature of the interventions, it was not possible to blind the therapist who treated the patients. Both groups received the concerned intervention for a period of four sessions per week for four weeks. The primary and secondary outcome measures were collected by a blinded physical therapist at baseline, 4 weeks, 8 weeks, and 6 months following the treatment.

### 2.3. Interventions

The concerned intervention procedures were provided by two orthopedic surgeons and two physical therapists. Following the corticosteroid injection, the recommended physical therapy was given for 4 weeks, after which the participants were asked to do their exercises at home for another 4 weeks. This was done by providing the patient with a hand-out, which included instructions regarding “dos and don’ts” while performing these exercises. They kept an exercise log book to enter their training activities during the study period.

#### 2.3.1. Corticosteroid Injection

A regular physical orthopedic examination was done by an orthopedic surgeon before the administration of an injection. Here, 1 mL triamcinolone acetonide (10 mg/mL) (Kenacort-A 10) with 1 mL lignocaine (1%) was administered into the most palpably tender point in the region of the lateral epicondyle [15]. To maintain participant blinding, the participants were not allowed to see the procedure of the administration of the injection. In addition, post-injection instructions were given to all participants by providing a printed brochure as well as explaining it to them personally. They were asked to take rest and not engage in strenuous activities for one week following the injection, even if they experience pain relief.

#### 2.3.2. Physical Therapy

After one week following the injection therapy, all of the participants were allowed to take regular physical therapy interventions by a licensed physical therapist with fifteen years of clinical experience in treating LE. The participants in both groups received physical therapy treatment for four sessions per week for four weeks and each session lasted for 30 to 40 min. To avoid intervention bias, a fixed physical therapy protocol was prepared based on recent evidence with the objectives of ameliorating pain and increasing functional activities and soft tissue healing. A therapist who had experience in operating an ESWT device provided the treatment to all participants in the ESWT active group. The treatment started with 250 “warm-up” pulses at 1.5 bar of air pressure, which acclimatized the participant to the ESWT treatment. Once the patient was comfortable with the treatment, the air pressure was increased to 2.5 bar, and 2000 pulses with a frequency of 8 Hz of dose were administered in the LE region. Then, with the same parameters, ESWT was applied to the trigger points of the extensor carpi radialis brevis muscle. This treatment was given once a week for four planned consecutive weeks [16]. For the placebo group, a special head that blocked the shockwaves from occurring was used, but was indistinguishable otherwise.

Progressive resistance exercises (PRE) were prescribed for the wrist extensors with a TheraBand based on the assessment of the individual muscles. In the early phase, the painful movements are trained with minimal resistance and then progress to the next level of resistance for the other joint movements. In the later phase, the progression of exercise intends to work on activity- or sports-specific rehabilitation. The therapist selected the exercise parameters (intensity, frequency, and duration) in every treatment session purely based on the individual capacities without exaggerating the symptoms [17]. Patient guidance was given through patient counselling on an individual basis and a pamphlet regarding the disease and home instructions was given to all the patients. The patients performed the home exercises daily for four weeks with eccentric exercise (30 repetitions, three times) and the isolated stretching of radial wrist extensors (three times daily for 30 s). The treatment adherence at home was monitored by a treating therapist before the commencement of every session by checking the exercise log book.

### 2.4. Outcome Measures

#### 2.4.1. Pain Intensity

The pain intensity was measured with a visual analog scale (VAS) and the participant was asked to note the perceived pain intensity on the 10 cm point scale, where scores ranged from “no pain” (0) to “worst imaginable pain” (10). VAS is considered a valid and reliable tool for measuring pain intensity in LE [18].

#### 2.4.2. Magnetic Resonance Imaging (MRI)

MRI has been established as a reliable and valid assessment tool to measure the extent of injury in LE patients. It was performed with a 3.0-T MR unit (Siemens Medical Solutions, Erlangen, Germany) with a flexible elbow coil (Philips, Eindhoven, The Netherlands). T2-weighted axial and coronal sections were taken and the extent of the tear was classified as low (<20%), intermediate (20–80%), and high grade (>80%) according to the percentage of injury [13].

#### 2.4.3. Ultrasound (US) Imaging

The ultrasound imaging was performed with a US unit (Esaote CA) with an 8–18 MHz linear array transducer. The stage of LE was classified as a high-grade tear (involves ≥ 50% of the tendon), low-grade tear (involves ≤ 50% of the tendon), suspected tendon tear (possible, but not evident tear), or no tendon tear [13].

#### 2.4.4. Functional Disability

The Patient-Rated Tennis Elbow Evaluation (PRTEE) questionnaire was used to measure the functional disability of the LE patients. The items are rated on an 11-point Likert scale and the disability is rated from 0—no disability to 100—significant functional disability. It is considered a valid and reliable tool to measure functional disability in LE [19].

#### 2.4.5. Handgrip Strength

The handgrip strength was measured with a handheld dynamometer and it is a reliable and valid measurement. The participant was asked to sit in a relaxed position with an elbow in a 90° flexed and pronated position. The participant was instructed to press the dynamometer with maximum effort and the measurements were taken. Three measurements were taken and the average of these was used for analysis [15].

#### 2.4.6. Patient Perception

Patient perception was measured using the Global Perceived Improvement questionnaire, which consists of a six-point Likert scale. It is a reliable and valid tool to measure patient perception related to LE [20].

#### 2.4.7. Kinesiophobia

The Tampa Scale for Kinesiophobia—adjusted version (TSK-AV) was used to measure the status of fear of injury. The scale consists of 13 items, which are marked on a four-point Likert scale. Obtaining a maximum score indicates more fear of injury and a lower score indicates less fear of injury [21].

#### 2.4.8. Depression

The Hospital Anxiety and Depression Scale (HADS) was used to measure the depression status of the LE patients. It consists of seven items each for depression and anxiety subscales. Scoring for each item ranges from 0 to 3, with 3 denoting the highest anxiety or depression level. A total subscale score of >8 points out of a possible 21 denotes considerable symptoms of anxiety or depression [22].

#### 2.4.9. Quality of Life

The EuroQol EQ-5D was used to measure the health-related quality of life, expressed as utility values ranging from 1 to 3, where 1 represents perfect health [23].

### 2.5. Sample Size

With a power of 0.8 and a significance level of 0.05, at least 30 participants were required for inclusion in each treatment arm (60 participants in total) to detect a clinically important mean difference between groups of four points on the VAS scores at 6 months, when assuming a standard deviation of one point and considering a 10% drop to follow-up. For other outcomes, we considered a between-group difference of 20% of the outcome measure’s scale to be clinically worthwhile.

### 2.6. Statistical Analysis

The study homogeneity was analyzed through the Kolmogorov–Smirnov test. The data analysis was performed on an intention-to-treat basis. For the missing data, the results obtained during the last available assessment of each participant were repeated. Analysis of variance with a linear mixed model was used to compare the effects of corticosteroid injection with ESWT between the active and placebo groups. The mean difference (MD) and 95% confidence interval (CI) were also calculated for each between-group comparison. The statistical analyses were processed using commercial statistical software (IBM SPSS—online version 20, Armonk, NY, USA) and a level of significance of *p* ≤ 0.05 was adopted for all tests.

## 3. Results

**Figure 1:**
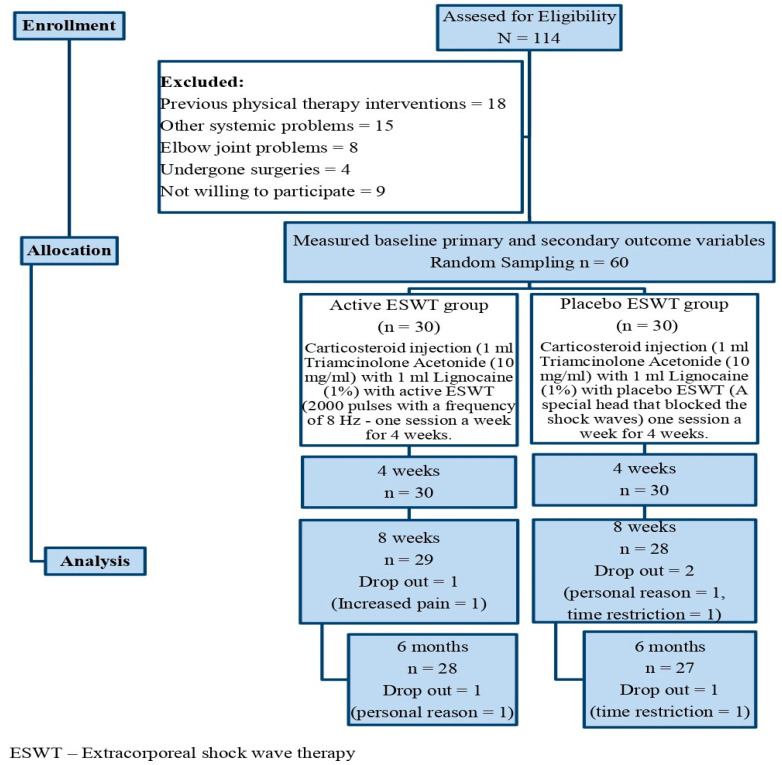
Flow chart showing the study details.

The flow of study participants throughout the trial is depicted in Figure 1 and their demographic characteristics were homogeneous and are described in Table 1. A total of 114 participants were screened, and 60 matched the selection criteria and were randomized into two groups. Five participants did not complete the follow-up measurement: two in the active ESWT group (one due to an increase in pain and the other due to personal reasons), and three in the placebo ESWT group (one due to personal reasons and two due to time restrictions).

**Figure 2:**
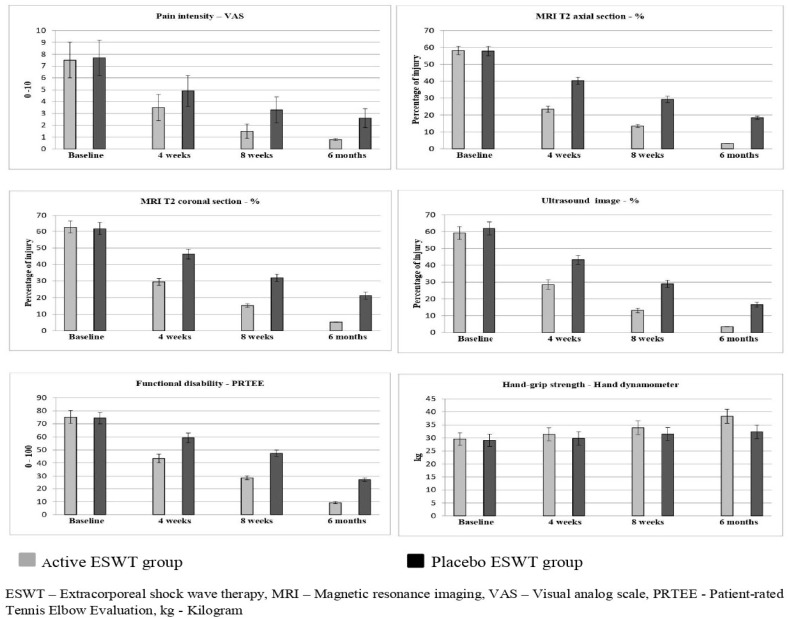
Pre- and post outcome measures of active and placebo ESWT groups.

The time and group (4 × 2) MANOVA of the primary variable (pain intensity—VAS) reports a statistically significant difference (*p* < 0.001) between the active and placebo groups. The post-intervention at 4 weeks (1.4; CI 95% 0.77 to 2.02) shows more improvement in the active group than in the placebo group. Improvements in the effects were noted after 8 weeks and at 6-month (1.8; CI 95% 1.50 to 2.09) follow-up (Table 2). The effect size of pain intensity (d = 1.88) shows a larger effect in the active ESWT group than in the placebo ESWT group. The time and group (4 × 2) MANOVA of other primary variables (MRI T2 axial and coronal section, ultrasound, functional disability and handgrip strength) report a statistically significant difference (*p* < 0.001) between group A and group B. The post-intervention measure of the percentage of injury through MRI and US imaging reports a significant statistical change (*p* < 0.001) at 4 weeks. Similar effects were noted for the 8-week and 6-month follow-up measurements (Figure 2). The same changes were noted in the functional disability and handgrip strength at 4 weeks, 8 weeks, and at the 6-month follow-up. The effect size of MRI T2 axial (d = 0.59), MRI T2 coronal CSA (d = 0.83), ultrasound (d = 4.05), functional disability (d = 1.05), and handgrip strength (d = 1.05) shows larger effects in the active ESWT group than in the placebo ESWT group.

**Figure 3:**
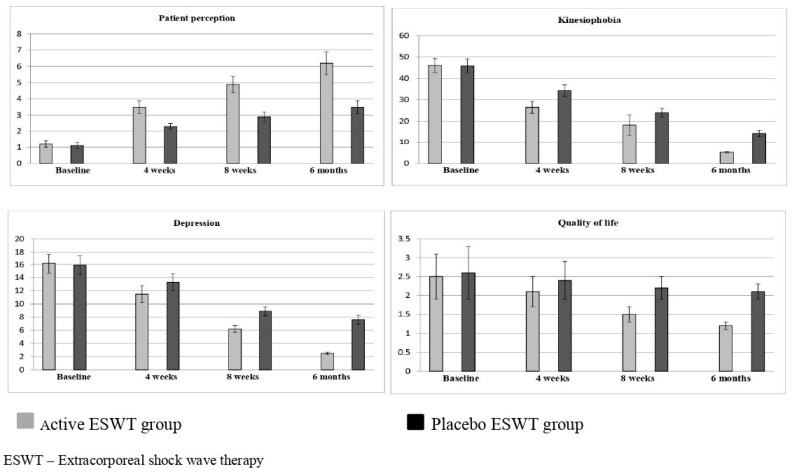
Pre- and post outcome measures of active and placebo ESWT groups.

The post-intervention at 4 weeks of all secondary variables shows more improvement in the active group than in the placebo group. Similar effects were noted after 8 weeks and at the 6-month follow-up. The scores show more significant changes (*p* < 0.001) in the active group than the placebo group, which are shown in Table 3. For the primary and secondary outcome measures, important between-group differences were detected. The graphical representations in Figure 2 and Figure 3 also show more improvements in all the variables in the active ESWT group than in the placebo ESWT group at various intervals.

Adverse effects:

No adverse reactions such as an increase in the signs and symptoms, excessive tissue injury, or tear enlargement after the intervention were noted. One participant from the active ESWT group mentioned an increase in pain after 8 weeks of treatment. He was discontinued from the study immediately and proper medical attention was given.

## 4. Discussion

The participants in the active ESWT group showed significant improvement compared to the placebo group at various intervals in all of the outcome measures. The positive changes in these groups show that the problem itself heals without any specific intervention. A study by Olaussen et al. found that corticosteroid (CS) injection with physiotherapy has a very good initial response in chronic LE [24]. Our reports are in agreement with Olaussen et al. and also found an added effect of extracorporeal shockwave therapy (ESWT) in LE. On the contrary, Coombes et al. observed that a combination of CS injection with physiotherapy provided no added benefit in LE on a long-term basis. They opposed using CS due to the high recurrence rates and adverse reactions at a later stage [25]. The injection of CS provides considerable pain reduction soon after administration, which promotes excessive use of the limb at an earlier stage [26]. Therefore, patients are instructed to keep the limb in a resting state for 2 days after the injection [27].

The therapeutically effective ESWT provided in this study consists of one session per week for four weeks, which involves the administration of 2000 pulses at a frequency of 8 Hz and a pressure of 2.5 bar, which were considered suitable parameters for treating chronic LE [28]. Gunduz et al. found that the choice of parameters for the treatment session should be considered according to the stage of the disease [29]. A study by Krol et al. stated that ESWT facilitates tissue healing, which leads to pain relief and considerable improvement in wrist function. They affirm that ESWT initiates a chain reaction, restoring the physiological function of the affected tissues [30].

In our study, we measured the extent and percentage of injury through MRI and US and found that active ESWT has significant improvements over placebo ESWT. Coel et al. found that the percentage of injury would associate with abnormal motion or compensation caused by injury [31]. Moreover, a significant improvement was also noted in the percentage of tissue injury in the placebo group, which might be due to the effects of the corticosteroid injection and the progressive resistance exercises. The pattern of improvement identified in the PRTEE scores suggests that progressive resistance exercise offered more rapid improvement. An increase in muscle strength improves the functional status [15] and psychological status of the participants. The use of a placebo ESWT combined with blinding is intended to prevent any bias resulting from non-specific effects associated with those receiving the intervention (placebo effects) [32].

There were a few dropouts and the adherence to the intervention was good. We chose the most apt statistical tests that would minimize Type 1 errors, since this is a non-serious and self-limiting condition. A few limitations were noted during the study. First, only male subjects were included in the study. Including both sexes would provide more information about these interventions. Second, a strict physiotherapy guideline prohibited an individual adjustment of treatment and may have influenced the results. Third, the study only includes chronic lateral epicondylitis patients, therefore it would be interesting to study the efficacy of ESWT in acute and sub-acute conditions using similar inclusion criteria to those in our study.

## 5. Conclusions

In conclusion, extracorporeal shockwave therapy has an added effect on corticosteroid injection in terms of reducing pain and improving tissue healing, functional disability, handgrip strength, patient perception, kinesiophobia, and depression status, as well as the health-related quality of life in people with chronic lateral epicondylitis. Future studies should examine different frequencies of extracorporeal shockwave therapy and different doses of corticosteroid injection in chronic lateral epicondylitis patients.

## Figures and Tables

**Table 1:** Demographic and clinical characteristics of active and placebo ESWT groups.

Sr. No	Variable	Active ESWT (*n* = 30)	Placebo ESWT (*n* = 30)	*p*-Value
1	Age (y)	47.12 ± 3.2	46.56 ± 4.1	n.s.
2	Height (m)	1.68 ± 0.16	1.67 ± 0.18	n.s.
3	Weight (kg)	68.8 ± 4.3	69.2 ± 4.8	n.s.
4	Side involved (%)			
	Right side	24 (80%)	23 (77%)	n.s.
	Left side	5 (17%)	6 (20%)	n.s.
	Bilateral	1 (4%)	1 (4%)	n.s.
5	Dominance side affected (%)			
	Dominance = Right	21/28 (75%)	21/27 (78%)	n.s.
	Dominance = Left	1/2 (50%)	1/3 (34%)	n.s.
6	Previous episodes of LE, N (%)	4/30 (13%)	4/30 (13%)	n.s.
7	Duration of pain (m)	4.1 ± 2.5	3.2 ± 2.9	n.s.
8	Education level			
	School level	2/30 (7%)	2/30 (7%)	n.s.
	Bachelor	18/30 (60%)	16/30 (53%)	n.s.
	Master	7/30 (23%)	10/30 (33%)	n.s.
	PhD	3/30 (10%)	2/30 (7%)	n.s.
9	Employment			
	Manual work	19/30 (63%)	21/30 (78%)	n.s.
	Non-manual work	7/30 (23%)	6/30 (20%)	n.s.
	Not working	4/30 (13%)	3/30 (10%)	n.s.
10	Percentage of tear			
	Low grade (less than 20%)	6/30 (20%)	5/30 (17%)	n.s.
	Intermediate grade (20–80%)	22/30 (73%)	23/30 (77%)	n.s.
	High grade (more than 80%)	2/30 (7%)	2/30 (7%)	n.s.

Numbers represent mean ± standard deviation (S.D.), n.s.—non-significant, ESWT—extracorporeal shockwave therapy, y—year, m—meter, kg—kilogram, LE—lateral epicondylitis, m—months.

**Table 2:** Pre- and post-primary outcome measures of active and placebo ESWT groups.

Sr. No	Variable	Duration	Active	Placebo	*p*-Value
1	Pain intensity—VAS (0–10 cm)	Baseline	7.5 ± 1.5	7.7 ± 1.5	0.607
		4 weeks	3.5 ± 1.1	4.9 ± 1.3	0.001 *
		8 weeks	1.5 ± 0.6	3.3 ± 1.1	0.001 *
		6 months	0.8 ± 0.09	2.6 ± 0.8	0.001 *
		*p*-value	0.001 *	0.001 *	
2	MRI T2 axial section—% (Percentage of injury)	Baseline	58.2 ± 2.5	57.9 ± 2.7	0.656
		4 weeks	23.5 ± 1.8	40.3 ± 2.1	0.001 *
		8 weeks	13.5 ± 0.8	29.3 ± 1.9	0.001 *
		6 months	3.2 ± 0.1	18.5 ± 0.9	0.001 *
		*p*-value	0.001 *	0.001 *	
3	MRI T2 coronal section—%(Percentage of injury)	Baseline	62.8 ± 3.8	61.9 ± 3.9	0.369
		4 weeks	29.5 ± 2.1	46.3 ± 2.9	0.001 *
		8 weeks	15.2 ± 1.1	32.0 ± 2.3	0.001 *
		6 months	5.2 ± 0.1	21.2 ± 2.1	0.001 *
		*p*-value	0.001 *	0.001 *	
4	Ultrasound image—%(Percentage of injury)	Baseline	59.2 ± 4.2	61.1 ± 4.1	0.081
		4 weeks	28.5 ± 2.8	43.3 ± 2.7	0.001 *
		8 weeks	13.2 ± 1.4	28.9 ± 2.1	0.001 *
		6 months	3.5 ± 0.1	16.6 ± 1.5	0.001 *
		*p*-value	0.001 *	0.001 *	
5	Functional disability PRTEE (0–100)	Baseline	75.2 ± 4.9	74.5 ± 4.5	0.566
		4 weeks	43.5 ± 3.4	59.3 ± 3.7	0.001 *
		8 weeks	28.7 ± 1.5	47.4 ± 2.5	0.001 *
		6 months	9.2 ± 0.7	27.3 ± 1.4	0.001 *
		*p*-value	0.001 *	0.001 *	
6	Handgrip strengthHand dynamometer (kg)	Baseline	29.6± 2.4	29.1± 2.4	0.423
		4 weeks	31.4± 2.5	29.8± 2.5	0.016 *
		8 weeks	33.9± 2.6	31.5± 2.5	0.001 *
		6 months	38.3± 2.7	32.3± 2.6	0.001 *
		*p*-value	0.001 *	0.001 *	

Numbers represent mean ± standard deviation (S.D.), * significant, ESWT—extracorporeal shockwave therapy, VAS—visual analog scale, MRI—magnetic resonance imaging, PRTEE—Patient-Rated Tennis Elbow Evaluation, kg—kilogram.

**Table 3:** Pre- and post-secondary outcome measures of active and placebo ESWT groups.

Sr. No	Variable	Duration	Active	Placebo	*p*-Value
1	Patient perception (GPI questionnaire)	Baseline	1.2 ± 0.2	1.1 ± 0.2	0.057
		4 weeks	3.5 ± 0.4	2.3 ± 0.2	0.001 *
		8 weeks	4.9 ± 0.5	2.9 ± 0.3	0.001 *
		6 months	6.2 ± 0.7	3.5 ± 0.4	0.001 *
		*p*-value	0.001 *	0.001 *	
2	Kinesiophobia (TSK-AV)	Baseline	46.1 ± 3.2	45.9 ± 3.3	0.812
		4 weeks	26.5 ± 2.8	34.3 ± 2.7	0.001 *
		8 weeks	18.2 ± 4.7	24.0 ± 2.0	0.001 *
		6 months	5.2 ± 0.5	14.2 ± 1.5	0.001 *
		*p*-value	0.001 *	0.001 *	
3	Depression (HADS)	Baseline	16.2 ± 1.5	15.9 ± 1.4	0.426
		4 weeks	11.5 ± 1.3	13.3 ± 1.3	0.001 *
		8 weeks	6.2 ± 0.5	8.9 ± 0.7	0.001 *
		6 months	2.5 ± 0.2	7.6 ± 0.7	0.001 *
		*p*-value	0.001 *	0.001 *	
4	Quality of life (EuroQol EQ-5D)	Baseline	2.5 ± 0.6	2.6 ± 0.7	0.554
		4 weeks	2.1 ± 0.4	2.4 ± 0.5	0.001 *
		8 weeks	1.5 ± 0.2	2.2 ± 0.3	0.001 *
		6 months	1.2 ± 0.1	2.1 ± 0.2	0.001 *
		*p*-value	0.001 *	0.001 *	

* Significant, ESWT—extracorporeal shockwave therapy, GPI—Global Perceived Improvement, TSK-AV—Tampa Scale for Kinesiophobia—adjusted version, HADS—Hospital Anxiety and Depression Scale, EuroQol EQ-5D—European Quality of Life—five dimensions.

## Data Availability

The data are not publicly available, but can be obtained from the corresponding author on request.
